# Bacterial Abundance, Diversity and Activity During Long-Term Colonization of Non-biodegradable and Biodegradable Plastics in Seawater

**DOI:** 10.3389/fmicb.2021.734782

**Published:** 2021-11-18

**Authors:** Charlene Odobel, Claire Dussud, Lena Philip, Gabrielle Derippe, Marion Lauters, Boris Eyheraguibel, Gaëtan Burgaud, Alexandra Ter Halle, Anne-Leila Meistertzheim, Stephane Bruzaud, Valerie Barbe, Jean-Francois Ghiglione

**Affiliations:** ^1^CNRS, UMR 7621, Laboratoire d’Océanographie Microbienne (LOMIC), Sorbonne Université, Observatoire Océanologique de Banyuls, Banyuls-sur-Mer, France; ^2^SAS Plastic@Sea, Observatoire Océanologique de Banyuls, Banyuls-sur-Mer, France; ^3^CNRS, UMR 6027, Institut de Recherche Dupuy de Lôme (IRDL), Université de Bretagne-Sud, Lorient, France; ^4^CNRS, UMR 6296, Institut de Chimie de Clermont-Ferrand (ICCF), Université Clermont Auvergne, Clermont-Ferrand, France; ^5^CNRS, EA 3882, Université de Brest, Laboratoire Universitaire de Biodiversité et d’Ecologie Microbionne (LUBEM), Plouzané, France; ^6^CNRS, UMR 5623, Laboratoire des Interactions Moléculaires et Réactivité Chimique et Photochimique (IMRCP), Université de Toulouse, Toulouse, France; ^7^CEA, CNRS, Génomique Métabolique, Genoscope, Institut François Jacob, Univ Evry, Université Paris-Saclay, Evry, France

**Keywords:** microbial ecotoxicology, plastisphere, biofouling, biofilm, plastic pollution

## Abstract

The microorganisms living on plastics called “plastisphere” have been classically described as very abundant, highly diverse, and very specific when compared to the surrounding environments, but their potential ability to biodegrade various plastic types in natural conditions have been poorly investigated. Here, we follow the successive phases of biofilm development and maturation after long-term immersion in seawater (7 months) on conventional [fossil-based polyethylene (PE) and polystyrene (PS)] and biodegradable plastics [biobased polylactic acid (PLA) and polyhydroxybutyrate-co-hydroxyvalerate (PHBV), or fossil-based polycaprolactone (PCL)], as well as on artificially aged or non-aged PE without or with prooxidant additives [oxobiodegradable (OXO)]. First, we confirmed that the classical primo-colonization and growth phases of the biofilms that occurred during the first 10 days of immersion in seawater were more or less independent of the plastic type. After only 1 month, we found congruent signs of biodegradation for some bio-based and also fossil-based materials. A continuous growth of the biofilm during the 7 months of observation (measured by epifluorescence microscopy and flow cytometry) was found on PHBV, PCL, and artificially aged OXO, together with a continuous increase in intracellular (^3^H-leucine incorporation) and extracellular activities (lipase, aminopeptidase, and β-glucosidase) as well as subsequent changes in biofilm diversity that became specific to each polymer type (16S rRNA metabarcoding). No sign of biodegradation was visible for PE, PS, and PLA under our experimental conditions. We also provide a list of operational taxonomic units (OTUs) potentially involved in the biodegradation of these polymers under natural seawater conditions, such as *Pseudohongiella* sp. and *Marinobacter* sp. on PCL, *Marinicella litoralis* and *Celeribacter* sp. on PHBV, or Myxococcales on artificially aged OXO. This study opens new routes for a deeper understanding of the polymers’ biodegradability in seawaters, especially when considering an alternative to conventional fossil-based plastics.

## Introduction

The marine environment has become sinks for vast quantities of anthropogenic debris, including mismanaged plastic waste, estimated to 4.8–12.7 million metric tons entering the oceans every year (Jambeck et al., 2015). Microplastics (< 5 mm) represent more than 90% of the total counts of marine plastic debris, mostly originated from larger debris fragmentation by a combination of physical, chemical, and biological processes (Eriksen et al., 2014). Microplastics are generally composed of polyethylene (PE), polypropylene (PP), and polystyrene (PS), three conventional polyolefins highly refractory to biodegradation because of their intrinsic features (Poulain et al., 2019). Issues related to marine plastic pollution are multifaceted and cross-sectoral, leading to numerous environmental, economic, and social impacts and thus calling for non-trivial legislative and non-legislative strategies, with some that have already been reported worldwide (Schnurr et al., 2018). Within the frame of transition to blue economy, a wide range of biodegradable plastics were developed as potential substitutes to conventional plastics (Science Advice for Policy by European Academies, 2020). Some are still petroleum-based, such as polycaprolactone (PCL) or oxobiodegradable plastics (OXO) made of PE with prooxidant additives stimulating abiotic oxidation processes needed for its biodegradation (Yashchuk et al., 2012; Eyheraguibel et al., 2017). Others are made from renewable resources including biological materials or agricultural resources (so called “bio-based”), such as polylactic acid (PLA) and polyhydroxybutyrate-co-hydroxyvalerate [PHBV, a commercially available polyhydroxyalkanoate (PHA)] (RameshKumar et al., 2020).

In the marine environment, the very diverse microbial communities living on any kind of plastic debris has been called “plastisphere” (Zettler et al., 2013). Microplastics offer a new habitat characterized by higher abundance and distinct communities as compared to the surrounding free-living or organic particle-attached bacteria (Dussud et al., 2018a). The colonization of new plastics released at sea until the formation of a mature biofilm has been described as a succession of several phases (Wright et al., 2020). During the first hours in marine waters, a “conditioning film” made of inorganic and organic matter supports the initial colonization phase generally initiated by Gammaproteobacteria and Alphaproteobacteria regardless of the polymer type and representing microorganisms with efficient surface adhesion (Oberbeckmann et al., 2015; Dussud et al., 2018b). A second phase consists of the biofilm growth involving a succession of rapidly growing bacteria such as Acidobacteria, Actinobacteria, Bacteroidetes, Cyanobacteria, Firmicutes, and Planctomycetes (Salta et al., 2013). The last phase that generally appears after 15 to 30 days in seawater relates to a mature biofilm and remains stable for several months (Kirstein et al., 2018). The description of these colonization phases has been done on several conventional plastic types, including on PE-based plastic bags (Lobelle and Cunliffe, 2011; Harrison et al., 2014), polyethylene terephthalate (PET)-based plastic bottles (Oberbeckmann et al., 2014), polyvinyl chloride (PVC) (Dang et al., 2008), PS coupons (Briand et al., 2012), and in comparisons between PE, PP, PET, and polycarbonate (PC) materials (Webb et al., 2009; De Tender et al., 2017). Fewer studies have compared conventional to biodegradable plastics (including PLA, PHBV, and OXO) in terms of community composition and dynamics (Eich et al., 2015; Dussud et al., 2018a; Cheng et al., 2021), with different experimental conditions and microbial parameters that made more challenging comparison between these studies.

In this study, we describe the colonization dynamics of microplastics made of conventional (PE and PS) and biodegradable polymers (PCL, PLA, and PHBV) in seawater. We also tested the initial formulation of OXO that is recalcitrant to biodegradation and different artificially aged OXO (AA-OXO) that can be further biodegraded by oxidative mechanisms in the environment. We hypothesized that mature biofilms from biodegradable plastics differed from conventional materials, both in terms of abundance, diversity, and activities. Such differences should permit the description of potentially biodegrading plastisphere members, which may differ according to the plastic type. We present here an original and comprehensive comparison of the bacterial colonization potential on six plastic formulations incubated in seawater by coupling heterotrophic production and ectoenzyme measurements, in parallel to bacterial abundance (epifluorescence microscopy and flow cytometry) and bacterial diversity (16S rRNA metabarcoding) during a long-term colonization in seawater (7 months).

## Materials and Methods

### Plastic Manufacturing Process

The first four polymer films were commercially available, made of low-density PE (Borealis, ref. FA6224, Austria), PS (Goodfellow, ref. 065-289-66, United States), PCL (CAPA 6800, Perstorp Company, Sweden), and PLA (Goodfellow, ref. 247-628-87, United States). The fifth film was formed from dried PHBV pellets (supplied by Tianan Biological Materials Co. Ltd., under the trade name ENMAT Y1000P, China) by using compression molded in a CarverR hydraulic press at 180°C under a pressure of 10 metric tons for 3 min. The sixth film was made of OXO with the same low-density PE as described above, but additivated with D_2_W formulation based on manganese and iron and extruded at 180°C using a laboratory scale Rondol linear 18 mm blown film (provided by Symphony Environmental Ltd., United Kingdom).

We also used two artificially aged OXO (AA-OXO) made of the same OXO as described above but differing by the exposure time to UV (according to ASTM D5208: UVA 340, 0.78 W m^–3^ nm^–1^) during 48 h for AA_48_OXO and during 144 h for AA_144_OXO. In both cases, UV aging was followed by a thermal aging during 96 h at 70°C (ASTM D5510) to increase the oxidation level. The same protocol was used for artificially aging of PE film by UV exposure for 144 h followed by a thermal aging during 96 h at 70°C, called AA_144_PE film hereafter. Artificial aging was stopped when reaching carbonyl index of 1x/100 for AA_48_OXO and 3x/100 for AA_144_OXO, where x was the film thickness. Carbonyl index values were determined as the ratio of the peak intensity at 1,715 cm^–1^ to the peak intensity at 1,460 cm^–1^ (Selke et al., 2015) by Fourier transform infrared spectroscopy (FTIR) analysis (Spectrum 100 equipped with an ATR attenuated total reflectance, Perkin-Elmer). The level of 1x/100 was previously demonstrated as a prerequisite for biodegradability of OXO, according to the French agreement Association Française de NORmalization (AFNOR AC t51-808, 2012). No sign of oxidation was observed for the AA_144_PE film by FTIR, which served as a control in our experiment.

The film’s thicknesses were 200 μm for PS, PHBV, PCL, and PLA and 100 μm for PE, AA_144_PE, OXO, AA_48_OXO, and AA_144_OXO. Each of the nine films was cut into 100 circular pieces of 9 mm diameter with a puncher machine. Polymer pieces were sterilized with 70% ethanol and rinsed three times with sterile seawater (SSW) before incubation in natural seawater.

### Incubation Under Natural Seawater Conditions During 7 Months

Each material type was incubated in aquariums with direct circulation to the sea, as previously described (Dussud et al., 2018a). Briefly, we used 10 identical aquariums consisting of trays of 1.8 L capacity (Sodispan, Spain) continually renewed with 20 μm pre-filtered seawater (Leroy Merlin, France) by individual water delivery valves and overflow ports setting a flow rate of 0.1 L min^–1^. Seawater was pumped at 30 m from the coast and 4 m depth in the Banyuls bay, close to the SOLA observatory station (NW Mediterranean Sea, France). During the experiment, seawater temperature in the aquariums and in the Banyuls bay was between 12.5 and 19.5°C and salinity remained stable at 38.5. Incubations in aquariums were processed in the dark to avoid UV-related degradation of the polymers and algal development. One aquarium was used as control and contained only circulating seawater. The nine other aquariums contained the pieces of each film (PE, AA_144_PE, OXO, AA_48_OXO, AA_144_OXO, PS, PHBV, PCL, and PLA). Plastic pieces of each material type were sampled after 3, 10, 31, 74, 116, and 206 days, together with seawater from the control aquarium.

### Epifluorescence Microscopy

One piece of each polymer was rinsed at each sampling time with sterilized seawater and fixed for 1 h at 4°C with 1% (v/v) glutaraldehyde (final concentration) before freezing. Epifluorescence microscopy observations were done using an Olympus AX70 PROVIS after 4′,6-diamidino-2-phenylindole (DAPI) staining according to Porter and Feig (1980). Pictures were taken on 10 fields of each polymer type and cell counting was done with the software Microbe Counter.

### Flow Cytometry

Three pieces of each polymer were rinsed at each sampling time with sterile seawater before cell detachment, as previously described (Dussud et al., 2018b). Briefly, cell detachment pre-treatment was performed using 1 mM pyrophosphate (30 min at room temperature in the dark) followed by a sonication step (3 × 5 s, 40 kHz, 30% amplitude, sterilized probe Branson SLPe). Cells were fixed for 1 h at 4°C with 1% (v/v) glutaraldehyde (final concentration) before freezing. In parallel, 3 × 1 ml of seawater from the control aquarium were also fixed using the same procedure. A 500 μl subsample was mixed with the nucleic acid dye SYBR Green I (final concentration 0.05% v/v, Sigma Aldrich) at room temperature for 15 min in the dark. Cell counts were performed with a FACSCanto II flow cytometer (BD Bioscience, San Jose, CA) equipped with a blue laser (488 nm, air-cooled, 20 mW solid state), as previously described (Mével et al., 2008). Cell counts were expressed as number of cells mm^–2^ for polymers and as number of cells ml^–1^ for seawater.

### Heterotrophic Bacterial Production

Bacterial production (BP) was measured for each polymer type and at each sampling time by ^3^H-leucine incorporation into proteins, as previously described (Dussud et al., 2018b). Briefly, ^3^H-leucine (specific activity 112 Ci mmol^–1^, Perkin Elmer) was added at 1 nM final concentration (completed with cold leucine to 150 nM) for polymer samples and 4.3 nM (completed with cold leucine to 42.8 nM) for 3 μm filtered seawater from the control aquarium. Triplicate samples were incubated in the dark at *in situ* temperature for 3 h. The polymers were rinsed with 5% TCA and 70% ethanol and then resuspended in 1.0 ml of a liquid scintillation cocktail (Ultima Gold). Radioactivity was determined in a Beckman Scintillation Counter (LS 5000CE) and data analyzed with the Microwin 2000 software. We used the empirical conversion factor of 1.55 ng C pmol^–1^ of incorporated leucine to calculate BP (Simon and Azam, 1989). Values were given in ng C mm^–2^ h^–1^ for plastics and in ng C ml^–1^ h^–1^ for seawater.

### Extracellular Enzymatic Activities

Three extracellular enzymatic activities were analyzed for amino peptidase, β-glucosidase, and lipase. Triplicates of 1/4 of plastic piece of each material type were placed in a 96-well plate with fluorogenic model substrates that were L-leucine-7amido-4-methyl coumarin (Leu-MCA, 10 mM final, Sigma Aldrich) for amino peptidase, 4-methylumbelliferyl-β-D-glucoside at 20 mM for β-glucosidase (MUF-β-Glc, 20 mM final, Sigma Aldrich), and 4-methylumbelliferyl-oleate for lipase (MUF-Oleate, 20 mM final, Sigma Aldrich). These saturated concentrations for amino peptidase, β-glucosidase and lipase and optimized time incubations were determined prior to the extracellular enzymatic activity measurement (data not shown). After 4 h (for seawater) or 8 h (for plastics) of incubation, 50 μl of a stop solution (SDS 10%) was added in all wells. Fluorescence was quantified using VICTOR3 spectrofluorometer (PerkinElmer) at a 380 nm wavelength for MCA and 364 nm for MUF substrates. A control was done in triplicate for each activity and each plastic type by adding the stop solution at the beginning of incubation. The spectrofluorometer was calibrated with MCA and MUF standard solutions diluted in sterile seawater. Values were given in nmol mm^–2^ h^–1^ for polymers and in nmol ml^–1^ h^–1^ for seawater.

### DNA Extraction, PCR Amplification, and Sequencing

Plastic pieces were sampled for all plastic types and at each sampling time with sterilized forceps and stored at −80°C until analysis. In parallel, 1 L seawater was sampled on the same day in the control aquarium, successively filtered onto 3-μm and 0.2-μm pore size polycarbonate filters (47 mm diameter, Nucleopore), and filters were stored at −80°C until analysis. DNA extraction was performed on polymers or seawater at all sampling time, using a classical phenol-chloroform method for seawater samples, modified for the plastic samples as previously described (Dussud et al., 2018b). Briefly, the modification consisted in a sonication pretreatment (3 × 5 s with 30% amplitude, sterilized probe Branson SLPe) in order to increase the detachment of biofilm forming bacteria. The molecular size and purity of the DNA extracts were analyzed by agarose gel electrophoresis (1%) and the DNA was quantified by spectrophotometry (GeneQuant II, Pharmacia Biotech). Primers for Polymerase Chain Reaction (PCR) amplification of the 16S V3–V5 region were 515F-Y and 926R, well-suited for marine samples according to Parada et al. (2016) with Illumina-specific primers and barcodes. Sequencing was performed on Illumina MiSeq by Genoscope (Evry, France). Raw FASTA files were deposited at EBI under the accession number PRJEB37662. Sequence analysis was conducted using the FROGs pipeline (Escudié et al., 2017) supported on the Galaxy instance of Toulouse Midi-Pyrenees bioinformatics platform. The pre-process tool was used to merge the paired-ends raw reads using Flash (Magoè and Salzberg, 2011) and quality filtering and primers trimming with cutadapt (Martin, 2011). Sequence clustering was performed with the Swarm algorithm (Mahé et al., 2014). Chimeras were detected with the VSEARCH algorithm, by *de novo* UCHIME method (Edgar et al., 2011; Rognes et al., 2016) and were removed. Clusters were assigned with the Silva 128 16S rRNA database (Quast et al., 2012) and clusters that did not belong to Bacteria kingdom were removed as well as chloroplast and mitochondrial sequences. The clusters that had a reads number abundance lower than 0.005% of all reads were removed, according to Bokulich et al. (2013). The number of sequences per sample was normalized by rarefaction (*n* = 17,865). Finally, a table with 66 samples and 1,517 clusters was obtained.

### Statistical Analysis

For each sample, we calculated Chao1 species richness estimator, Simpson and Shannon diversity indexes, and Pielou’s evenness using the Phyloseq package (McMurdie and Holmes, 2013) of R software suite (Table 1). The non-parametric estimator of Chao1 was calculated as follows: S = S_obs_ + a^2^/2b ^∗^ (N − 1)/N, where S_obs_ is the number of OTUs observed in the sample, N is the number of sequences per sample, a is the number of OTUs detected only once, and b is the number of OTUs detected only twice (Chiu et al., 2014). Simpson diversity index was computed as D = 1 −Σ[N_i_^∗^(N_i_ − 1)/N^∗^(N − 1)], where N_i_ is the abundance of the i^th^ OTU in the sample (Simpson, 1949). Shannon index was calculated as H = -Σ[p_i_^∗^log(p_i_)], where p_i_ is the proportion of the i^th^ OTU (Shannon and Weaver, 1949) and Pielou’s evenness index was R = H/log_2_S_obs_ (Pielou, 1966). Differences with incubation time and between biodegradable and non-biodegradable plastics in richness and diversity indexes were tested using an ANOVA test (for normally distributed data, determined by a Shapiro test) or a Kruskal–Wallis test with R software.

**TABLE 1:** OTUs richness (Observed) and diversity indexes (Chao1, Pielou, Shannon and Simpson) of free-living (F), organic particle-attached bacteria (A) and biofilms of different plastic types (PE, AA_144_PE, OXO, AA_48_OXO, AA_144_OXO, PS, PHBV, PCL, PLA) according to immersion time in seawater (3, 10, 31, 74, 116 and 206 days).

Sample type	Day	Observed	Chao1 ± se	Pielou	Shannon	Simpson
F	3	445	568 ± 27	0.67	4.11	0.95
	10	535	742 ± 40	0.66	4.16	0.94
	31	696	913 ± 40	0.67	4.40	0.95
	74	505	693 ± 38	0.65	4.07	0.94
	116	376	545 ± 40	0.62	3.69	0.91
	206	235	321 ± 27	0.53	2.92	0.89
A	3	810	946 ± 28	0.74	4.95	0.98
	10	905	1001 ± 21	0.80	5.47	0.98
	31	901	982 ± 19	0.86	5.86	0.99
	74	897	979 ± 19	0.80	5.46	0.99
	116	793	872 ± 19	0.81	5.40	0.99
	206	453	677 ± 51	0.68	4.13	0.96
PE	3	226	336 ± 31	0.51	2.78	0.90
	10	404	532 ± 31	0.58	3.48	0.90
	31	938	1041 ± 23	0.82	5.63	0.99
	74	738	879 ± 27	0.63	4.18	0.93
	116	412	486 ± 20	0.54	3.23	0.88
	206	777	871 ± 21	0.78	5.20	0.99
AA_144_PE	3	286	375 ± 25	0.56	3.16	0.91
	10	433	561 ± 32	0.66	4.01	0.95
	31	882	1000 ± 24	0.80	5.44	0.99
	74	592	681 ± 22	0.72	4.57	0.96
	116	599	654 ± 15	0.75	4.83	0.98
	206	716	854 ± 30	0.75	4.96	0.98
OXO	3	240	344 ± 30	0.54	2.96	0.89
	10	479	607 ± 28	0.71	4.40	0.98
	31	702	909 ± 37	0.64	4.22	0.96
	74	611	691 ± 21	0.72	4.64	0.97
	116	559	599 ± 12	0.77	4.88	0.98
	206	569	729 ± 32	0.57	3.60	0.91
AA_48_OXO	3	182	225 ± 16	0.51	2.66	0.86
	10	375	485 ± 26	0.62	3.70	0.94
	31	420	530 ± 26	0.52	3.12	0.85
	74	501	601 ± 25	0.70	4.36	0.96
	116	528	609 ± 21	0.72	4.50	0.97
	206	639	777 ± 29	0.72	4.65	0.97
AA_144_OXO	3	183	254 ± 26	0.50	2.61	0.82
	10	519	665 ± 30	0.59	3.70	0.94
	31	812	935 ± 25	0.74	4.94	0.97
	74	519	654 ± 31	0.71	4.42	0.96
	116	486	573 ± 22	0.68	4.23	0.95
	206	352	557 ± 51	0.61	3.56	0.93
PS	3	503	682 ± 35	0.53	3.33	0.90
	10	488	647 ± 32	0.57	3.55	0.93
	31	666	791 ± 26	0.61	3.95	0.92
	74	674	868 ± 35	0.56	3.62	0.88
	116	825	902 ± 18	0.80	5.38	0.99
	206	830	939 ± 25	0.85	5.74	0.99
PHBV	3	260	332 ± 20	0.43	2.39	0.79
	10	388	541 ± 37	0.67	3.98	0.96
	31	317	471 ± 38	0.55	3.19	0.92
	74	609	835 ± 46	0.70	4.51	0.97
	116	535	667 ± 29	0.68	4.30	0.97
	206	343	430 ± 25	0.69	4.00	0.96
PCL	3	478	631 ± 31	0.51	3.12	0.89
	10	271	452 ± 47	0.54	3.04	0.91
	31	738	853 ± 23	0.64	4.24	0.94
	74	357	495 ± 32	0.58	3.44	0.92
	116	370	511 ± 34	0.53	3.12	0.87
	206	158	248 ± 29	0.33	1.67	0.67
PLA	3	223	327 ± 29	0.47	2.53	0.88
	10	665	815 ± 29	0.70	4.52	0.97
	31	739	884 ± 28	0.65	4.31	0.94
	74	793	887 ± 21	0.76	5.11	0.98
	116	840	944 ± 24	0.84	5.62	0.99
	206	785	908 ± 26	0.75	5.02	0.98

Unweighted-pair group method with arithmetic mean (UPGMA) dendrogram based on Bray-Curtis dissimilarities was used for visualization of beta-diversity. A series of dissimilarity profile permutation tests (SIMPROF, PRIMER 6) was performed under a simple null hypothesis of no meaningful structure within a sub-cluster. Significant branches (SIMPROF, *p* < 0.05, in black in Figure 4) were used to define sample clusters. One-way analysis of similarity was performed on the same distance matrix (ANOSIM, PRIMER 6) to test the significant difference and to partition the variance of the beta-diversity matrix between days of incubation and between polymer types and seawater. Similarity Percentage analysis (SIMPER) was performed to identify the contribution of each operational taxonomic unit (OTU), using PRIMER 6. A first comparison between PE and AA_144_PE revealed no specific species contribution of the plastisphere of one polymer to the other. The analyses then focused on the contributions of PE compared to other polymers (PHBV, PCL, OXO, AA_48_OXO, AA_144_OXO, AA_144_PE, PS, and PLA) at final time (D206). A threshold at 50% was applied to identify the most relevant species. For each OTU identified, in order to improve the affiliation attributed by Silva 128 16S rRNA database, the sequence was blasted to the NCBI databases “16S ribosomal RNA from bacteria and archaea” and “nt.” In some cases and depending on the identity percentage of the matches, a new affiliation has been proposed (Table 2). The data and graphical representations were performed using R statistical software version 3.5.0 (R Core Team, 2018).

**TABLE 2:** Taxonomy, relative abundance (r.a.) and contribution (cb) details (in %) of the 41 OTUs identified by SIMPER analysis, up to 30% of cumulative dissimilarity between PE and the other 8 plastics materials (AA_144_PE, OXO, AA_48_OXO, AA_144_OXO, PS, PHBV, PCL, PLA) after 206 days of immersion in seawater.

	AA_144_PE		OXO		AA_48_OXO		AA_144_OXO		PS		PHBV		PCL		PLA	
	
OTU taxonomy	r.a.	cb	r.a.	cb	r.a.	cb	r.a.	cb	r.a.	cb	r.a.	cb	r.a.	cb	r.a.	cb
*Marinicella litoralis*	0	0	0	0	0	0	0	0	0.017	0	5.9	3.2	0	0	0	0
Oceanospirillales	3.0	1.6	0.17	0	0.10	0	0.034	0	0.18	0	0	0	0	0	0.37	0
Oceanospirillales	4.5	3.5	0.20	0	0.067	0	0.017	0	0.19	0	0	0	0	0	0.25	0
*Pseudohongiella* sp.	0	0	0	0	0	0	0	0	0	0	0	0	52	26	0.011	0
*Polycyclovorans* sp.	7.9	3.4	4.7	0	7.6	0	0.022	0	0.43	0	0	0	0.062	0	5.7	0
Thioprofundaceae	0.44	0	0.12	0	0.067	0	0.43	0	2.5	1.8	0.12	0	0	0	0.68	0
Chromatiaceae	0.011	0	0.0056	0	0	0	0	0	0.034	0	0	0	0.056	0	3.7	2.8
Chromatiales	1.3	0	0.13	0	0	0	0	0	0	0	0	0	0	0	0	0
*Spongiibacter* sp.	0.23	0	0.22	0	0.52	0	0.011	0	0.062	0	0	0	0.073	0	4.5	0
Cellvibrionaceae	0	0	0	0	0.090	0	12	6.8	0.0056	0	0	0	1.2	0	0.15	0
Arenicellaceae	0.011	0	0	0	0.056	0	0.63	0	0.0056	0	0	0	0	0	0.017	0
Arenicellaceae	3.9	2.6	0.85	0	1.3	0	1.3	0	1.5	0	0.045	0	0	0	0.20	0
*Pseudoalteromonas* sp.	0	0	0	0	0	0	0	0	2.5	2.2	0	0	0	0	0	0
*Marinobacter* sp.	0	0	0	0	0	0	0	0	0	0	0	0	19	9.4	0.44	0
Gammaproteobacteria	1.8	1.3	14	9.9	5.0	2.8	0.017	0	0.15	0	0	0	0	0	0.76	0
Gammaproteobacteria	0.13	0	0.050	0	0.028	0	8.0	4.5	0.77	0	2.8	0	0	0	0.34	0
Gammaproteobacteria	3.0	3.4	1.9	0	0.062	0	0.12	0	0.42	0	0	0	0	0	0.017	0
Gammaproteobacteria	0	0	0	0	0	0	0.0056	0	0	0	9.7	5.2	0.0056	0	0	0
Gammaproteobacteria	0.36	0	0.084	0	0.10	0	0.0056	0	2.3	1.8	0	0	0	0	0.24	0
Myxococcales	0	0	0	0	0.16	0	10	5.7	0.011	0	0	0	0	0	0	0
*Desulfovibrio* sp.	0	0	0	0	0	0	0	0	0	0	0	0	0	0	3.1	2.5
*Nitrospira* sp.	0.57	0	0.17	0	0.022	0	0.028	0	1.9	1.4	0	0	0	0	0.63	0
*Celeribacter* sp.	0	0	0	0	0	0	0	0	0	0	3.4	1.8	0	0	0	0
*Marivita* sp.	0	0	15	11	3.4	2.6	0.017	0	0.039	0	0	0	0	0	0.011	0
Rhodobacteraceae	0.31	0	0.050	0	0.88	0	0.0056	0	0.073	0	0	0	0	0	0.16	0
Rhodobacteraceae	0.050	0	0.045	0	0.27	0	0.039	0	0.10	0	8.9	4.8	0.38	0	1.4	0
Rhodobacteraceae	0.034	0	0.011	0	0.0056	0	0.028	0	0.11	0	4.4	2.3	0	0	0.13	0
Hyphomonadaceae	1.1	0	0.11	0	0.16	0	0	0	0.078	0	0	0	0	0	4.8	3.3
Hyphomonadaceae	0	0	0.011	0	3.2	2.4	0.0056	0	0.011	0	0.0056	0	0	0	0.0056	0
Rhodobacterales	0.017	0	20	15	6.3	4.7	0.0056	0	0.034	0	0	0	0	0	0.022	0
Rhizobiales	0	0	0	0	7.5	5.7	0	0	0	0	0	0	0	0	0.039	0
Selenomonadales	0	0	0	0	0	0	0	0	0	0	0	0	0	0	4.7	3.8
Clostridiales	0	0	0	0	0	0	0	0	0	0	0	0	0	0	4.8	3.9
Saprospirales	5.1	3.0	1.8	0	0.39	0	0.0056	0	1.1	0	0.022	0	0	0	0.20	0
Saprospirales	2.7	0	0.20	0	0.078	0	0	0	0.57	0	0	0	0	0	0.050	0
Saprospirales	1.2	0	0.49	0	0.034	0	0	0	0.16	0	0.0056	0	0	0	0.0056	0
Flavobacteriaceae	0.15	0	0.13	0	1.7	0	17	9.8	0.067	0	1.7	0	0	0	0.028	0
Flavobacteriaceae	0	0	0	0	0	0	0	0	0	0	8.2	4.4	0.0056	0	0.0056	0
Cryomorphaceae	0.19	0	0.073	0	0.050	0	0	0	0	0	0	0	0	0	4.8	3.7
Acidobacteria	3.2	0	0.82	0	0.45	0	0.045	0	0.70	0	0.11	0	0	0	0.034	0
Flavobacteriaceae	0	0	0	0	0	0	0	0	0	0	0	0	16	8.0	0.0056	0

## Results

### Visual Changes of the Materials

**FIGURE 1:**
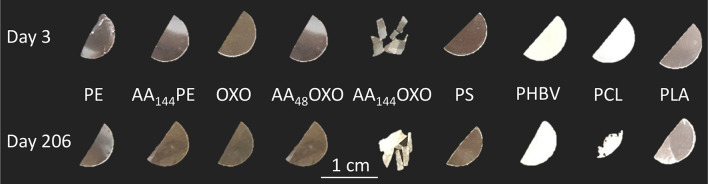
Plastic pieces of different composition (PE, OXO, PS, PHBV, PCL, and PLA) at the beginning (3 days) and at the end (206 days) of the experimental immersion in seawater. A pretreatment by artificial aging was applied for PE and OXO films by UV-A exposure during 48 h (AA_48_) or 144 h (AA_144_) followed by a thermal aging during 96 h at 70°C.

After 3 days of immersion in seawater, we observed that the translucent PHBV fragment whitened and that AA_144_OXO was very breakable, whereas the other plastic pieces showed no visual changes (Figure 1). The only change after 206 days in seawater was observed for PCL pieces, which showed a fivefold reduction in size compared to day 3.

### Epifluorescence Microscopy and Flow Cytometry

**FIGURE 2:**
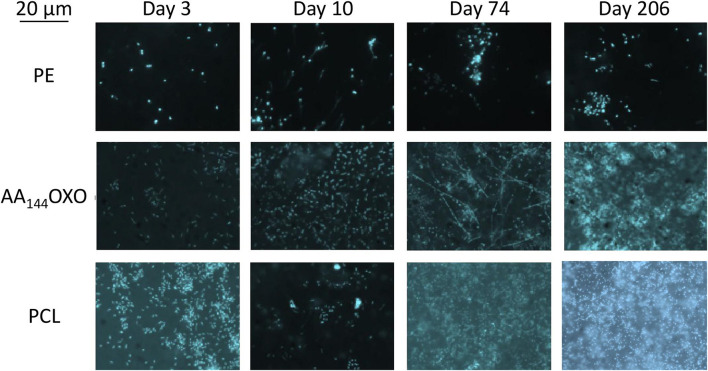
Epifluorescence micrographs of a selection of DAPI-stained plastics (PE, AA_144_OXO, and PCL) showing the formation and evolution of the biofilms of after 3, 10, 74, and 206 days of immersion in seawater. Artificial aging (AA) pretreatment applied for OXO polymers consisted in UV-A exposure during 144 h (AA_144_) followed by a thermal aging during 96 h at 70°C.

Epifluorescence microscopy showed the successive steps of colonization and maturation of the biofilm during 206 days in seawater (Figure 2). No or very few cells were detected on plastics after their sterilization and before their immersion in seawater. The presence of a mature biofilm together with extracellular polymeric substances was visible in all plastic types at the end of the experiment, but biofilms presented higher thickness for PCL and AA_144_OXO compared to the other plastics. Counting was not possible for PHBV because of autofluorescence background signal of the polymer.

**FIGURE 3:**
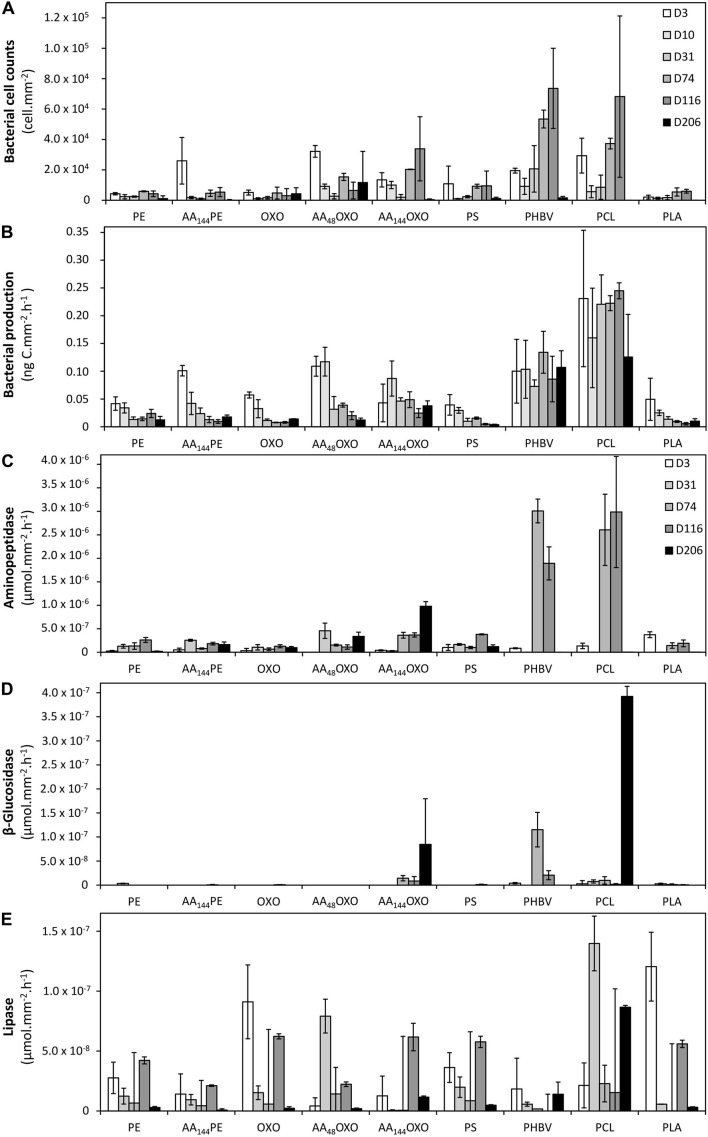
Bacterial cell counts **(A)**, bacterial heterotrophic production **(B)**, and bacterial extracellular aminopeptidase **(C)**, β-glucosidase **(D)**, and lipase activities **(E)** on the different plastic types (PE, AA_144_PE, OXO, AA_48_OXO, AA_144_OXO, PS, PHBV, PCL, and PLA) immersed in seawater for 3, 10, 31, 74, 116, and 206 days. No data available on day 10 for extracellular activities. Error bars indicate standard deviation.

It is noteworthy that no or very few cells were counted by flow cytometry after the sterilization of the plastic films and before their immersion in seawater, as found by epifluorescence microscopy. Flow cytometry confirmed the succession of phases during the colonization of the plastics (Figure 3A). The primo-colonization was characterized by a relatively high number of cell counts at day 3 (mean = 1.6 × 10^4^, SD = 1.1 × 10^4^ cells mm^–2^, *n* = 9) that decreased at day 10 (mean = 4.6 × 10^3^, SD = 1.1 × 10^3^ cells mm^–2^, *n* = 9), whatever the polymer type. Interestingly, a clear difference was found between PE and OXO (mean = 4.7 × 10^3^, SD = 5.6 × 10^2^ cells mm^–2^, *n* = 9) and the same polymer but artificially aged (one order of magnitude higher for AA_144_PE, AA_48_OXO, and AA_144_OXO).

A growing phase was found from day 10 to day 31 in all plastic films, followed by a stabilization phase of the biofilm that became mature and remains unchanged from day 31 to day 206 for PE, AA_144_PE, OXO, AA_48_OXO, PS, and PLA. Exceptions were found for PCL, PHBV, and AA_144_OXO that showed a continuous growth of biofilm until the end of the experiment (up to 2.0 × 10^4^ cells mm^–2^ after 116 days for PHBV, PCL, and AA_144_OXO). From 116 days in seawater, significant differences were found between cell counts on PHBV, PCL, and AA_144_OXO compared to other plastics (*t*-test, *p*-value < 0.05). The cell detachment treatment was less efficient after 116 days for PHBV, PCL, and AA_144_OXO, since we observed much lower cell counts by flow cytometry while epifluorescence microscopy confirmed the presence of abundant biofilms after 206 days. Control seawater cell counts remained stable during the entire experiment (mean = 2.3 ± 1.4 × 10^5^ cells ml^–1^).

### Bacterial Activities

#### Bacterial Heterotrophic Production

A clear distinction between biodegradable and non-biodegradable plastics was observed by cell incorporation of radiolabeled ^3^H-leucine (Figure 3B). The primo-colonizers on PE, AA_144_PE, OXO, PS, and PLA showed high heterotrophic activities during the first 3 days of immersion (between 3.94 × 10^–2^ ng C mm^–2^ h^–1^ and 1.01 × 10^–1^ ng C mm^–2^ h^–1^) followed by a dramatic and continuous decrease until the end of the experiment (between 3.87 × 10^–3^ and 1.77 × 10^–2^ ng C mm^–2^ h^–1^ at day 206). AA_48_OXO exhibited the same dynamics with a slight delay in time, with high activities until day 10, followed by a sharp decrease. The three other materials (PCL, PHBV, and AA_144_OXO) also presented high heterotrophic activities at the primo-colonization phase (day 3), but differed from the other plastics since their activities remained high and relatively stable until the end of the 206 days of seawater immersion. Maximum activities were found for PCL (2.45 × 10^–1^ ng C mm^–2^ h^–1^), followed by PHBV (1.34 × 10^–1^ ng C mm^–2^ h^–1^) and AA_144_OXO (8.69 × 10^–2^ ng C mm^–2^ h^–1^). Bacterial heterotrophic production remained stable during the entire experiment in control seawater (mean = 67.6 ± 42.2 ng C L^–1^ h^–1^).

#### Extracellular Enzymatic Activities

Similar trends were found when observing the extracellular aminopeptidase, β-glucosidase, and lipase activities (Figures 3C–E, respectively). The general trend found for bacterial heterotrophic production (see *Section 3.3.1*) of a high activity of the primo-colonizers (day 3; mean = 9.5 × 10^–5^ ± 3.3 × 10^–5^ μmol mm^–2^ h^–1^, mean = 7.1 × 10^–7^ ± 8.8 × 10^–7^ μmol mm^–2^ h^–1^, and mean = 3.8 × 10^–5^ ± 1.8 × 10^–5^ μmol mm^–2^ h^–1^ for aminopeptidase, β-glucosidase, and lipase, respectively) followed by a drastic decrease and further stabilization until the end of the experiment (206 days) was also found for extracellular activities. It was particularly true for lipase activities on PE, AA_144_PE, OXO, AA_48_OXO, PS, and PLA biofilms. Aminopeptidase activities (mean = 3.75 × 10^–4^ ± 8,95 × 10^–5^ μmol mm^–2^ h^–1^ with *n* = 30 for all plastics) and β-glucosidase (mean = 1.49 × 10^–5^ ± 4.49 × 10^–6^ μmol mm^–2^ h^–1^ with *n* = 30 for all plastics) remained low for these plastics during the entire experiment. Again, PCL, PHBV, and AA_144_OXO showed different trends when compared to other plastics. The highest extracellular activities were found when the biofilms became mature (after 31 days), with higher activities always found on PCL (3.0 × 10^–3^ ± 1.2 × 10^–3^ μmol mm^–2^ h^–1^, 3.9 × 10^–4^ ± 2.1 × 10^–5^ μmol mm^–2^ h^–1^, and 1.4 × 10^–4^ ± 2.3 × 10^–5^ μmol mm^–2^ h^–1^ for aminopeptidase, β-glucosidase, and lipase, respectively) and PHBV (3.0 × 10^–3^ ± 2.5 × 10^–4^ μmol mm^–2^ h^–1^, 1.1 × 10^–4^ ± 3.6 × 10^–5^ μmol mm^–2^ h^–1^, and 1.8 × 10^–5^ ± 2.6 × 10^–5^ μmol mm^–2^ h^–1^ for aminopeptidase, β-glucosidase, and lipase, respectively), followed by AA_144_OXO (9.8 × 10^–4^ ± 1.0 × 10^–4^ μmol mm^–2^ h^–1^, 8.5 × 10^–5^ ± 9.5 × 10^–5^ μmol mm^–2^ h^–1^, and 6.2 × 10^–5^ ± 1.1 × 10^–5^ μmol mm^–2^ h^–1^ for aminopeptidase, β-glucosidase, and lipase, respectively). On the other hand, extracellular enzymatic activities remained stable during the entire experiment in control seawater (1.1 × 10^–2^ ± 8.6 × 10^–3^ μmol L^–1^ h^–1^, 5.3 × 10^–5^ ± 1.2 × 10^–4^ μmol L^–1^ h^–1^, and 1.9 × 10^–3^ ± 3.5 × 10^–3^ μmol L^–1^ h^–1^ for aminopeptidase, β-glucosidase, and lipase, respectively).

### Dynamics of Bacterial Diversity

#### Alpha- and Beta-Diversity

Illumina Miseq DNA sequencing generated 9,054,841 paired sequences for the 66 samples, failing into 1517 OTUs after randomly resampling to 17,865 sequences per sample to provide statistical robustness when comparing diversity among samples.

The evolution of the Alpha-diversity was assessed with Chao1, Pielou, Shannon, and Simpson indexes (Table 1). Whatever plastic types, all indexes increased significantly between the primo-colonization (day 3) and the maturation phase of the biofilm (day 31) (pairwise *t*-test, *p* < 0.05). Chao1 species richness estimator almost doubled for all plastics between the primo-colonization and the maturation phase of the biofilm (mean = 390, SD = 135, *n* = 9 at day 3 and mean = 846, SD = 184, *n* = 9 at day 31) (Table 1). This index remained relatively stable until the end of the experiment for PE, AA_144_PE, OXO, AA_48_OXO, PS, and PLA biofilms, while after day 74 and up to day 206, a decrease was observed on PCL, PHBV, and AA_144_PE biodegradable polymers (mean = 753, SD = 147, *n* = 3 at day 31 and mean = 411, SD = 155, *n* = 3 at day 206). Shannon diversity index showed similar trends, with the exception of the decrease observed on the biodegradable polymers after day 74 and up to day 206, which was less on AA_144_PE and PHBV compared to PCL (Table 1). Bacterial diversity found in seawater on both free-living and organic particle-attached fractions was much less influenced by the temporal dynamics, where all Alpha-diversity indexes remained relatively stable during the entire course of the experiment (Shannon mean = 3.9, SD = 0.5, *n* = 6 on free-living and mean = 5.2, SD = 0.6, *n* = 6 on organic particle-attached fractions).

**FIGURE 4:**
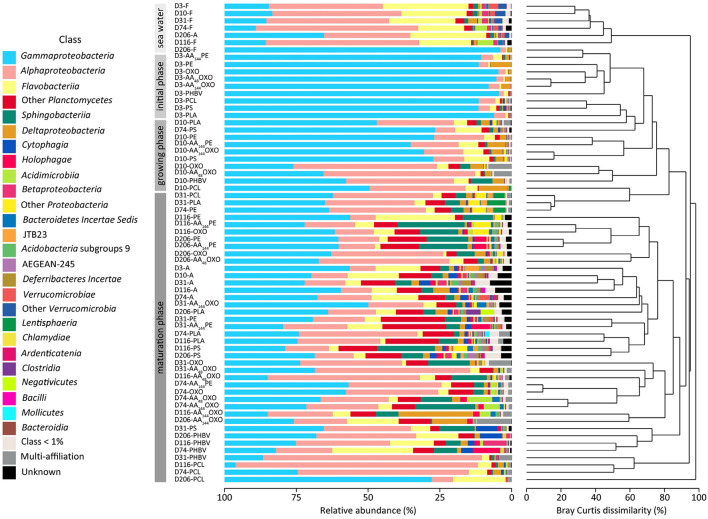
Temporal variation of taxonomic abundances and community structure of free-living (F), organic particle-attached bacteria (A) and biofilms of different materials (PE, AA_144_PE, OXO, AA_48_OXO, AA_144_OXO, PS, PHBV, PCL, and PLA) immersed in seawater for 3, 10, 31, 74, 116, and 206 days (D), by cumulative bar charts comparing taxonomic relative abundances (left) and by UPGMA dendrogram based on Bray–Curtis dissimilarities between 16S rRNA-based sequencing profiles (right).

Beta-diversity analysis showed strong dissimilarity (> 90%) between the free-living bacterial communities in seawater compared to plastic samples (Figure 4). Organic particle-attached bacterial communities in seawater also formed a separated cluster, with closer similarity with plastic samples, and especially with the non-biodegradable polymers (see below). Both free-living and organic particle-attached bacterial communities remained stable during the course of the experiment, which contrasted with the succession of distinct communities found on plastics. First, bacterial communities of all plastic materials clustered together during the primo-colonization phase (day 3), with little but significant changes observed on the biofilm growing phase (ANOSIM *R* = 0.622, *p* = 0.001 between day 3 and day 10). Second, we found a clear distinction between the mature biofilms (after 31 days) found on non-biodegradable (PE, AA_144_PE, OXO, AA_48_OXO, PS, and PLA) compared to biodegradable polymers (PHBV, PCL, and AA_144_OXO) (ANOSIM *R* = 0.287, *p* = 0.001). In particular, PCL and PHBV clusters strongly differed from the mature biofilms on other plastics, and AA_144_OXO formed a distinct cluster together with AA_48_OXO especially after 116 days of immersion in seawater.

#### Taxonomic Composition

Taxonomic analyses confirmed the specificity of the plastisphere compared to seawater free-living bacteria, the latter being dominated by Alphaproteobacteria (40%, mainly SAR11 clade with 24.8%), followed by Flavobacteriia (18.5%) and Gammaproteobacteria (28%, mainly Oceanospirillaceae with 7.9%) (Figure 4). Organic particle-attached bacterial communities were also dominated by Gammaproteobacteria (34.9%, mainly *Spongiibacter* sp. 4.3%), followed by Alphaproteobacteria (16%), Flavobacteriia (15%, mainly *Luteibaculum* sp. 5.3%), and Planctomycetacia (5.6%).

Whatever the plastic type, primo-colonizers (day 3) together with bacteria of the biofilm growing phases (day 10) belonged to Alteromonadales (mean = 21% SD = 11, *n* = 18), Oceanospirillales (mean = 17% SD = 14, *n* = 18, mainly represented by *Oleibacter* sp. and *Alcanivorax* sp.), and Cellvibrionales (mean = 10.1% SD = 9.4, *n* = 18, mainly represented by *Aestuariicella* sp.). Some exceptions were found for PCL (exhibiting higher proportions of *Oleiphilus* sp.), PLA (higher proportion of *Neptuniibacter* sp.), PS, and AA_144_OXO (dominated by *Spongiibacter* sp.) (Figure 4).

The mature biofilms (after 31 days) differed between material types. The non-biodegradable materials (PE, AA_144_PE, OXO, AA_48_OXO, and PS) together with PLA shared high proportions of Gammaproteobacteria (mean = 34 ± 11%), Alphaproteobacteria (mean = 24 ± 16%), Sphingobacteriia (mean = 8.8 ± 6.2%), and Flavobacteriia (mean = 5.0 ± 5.6%). Gammaproteobacteria class is composed of Cellvibrionales (mean = 13 ± 9%), with *Spongiibacter* sp. and *Zhongshania* sp. as main representatives as well as Xanthomonadales (mean = 5.6 ± 5.4%) with *Polycyclovorans* sp. PE and AA_144_PE showed increasing proportions of Saprospiraceae (up to 23%), Planctomycetes (up to 14%, mainly OM190 clade and *Planctomyces* sp.), and Arenicellaceae (up to 7%, mainly *Arenicella* sp.) over time. The same tendency was observed for OXO and AA_48_OXO with Rhodobacteraceae (up to 16%) and Flavobacteriaceae (up to 5%, mainly *Gilvibacter* sp.). Interestingly, AA_48_OXO was distinguished by large proportions of Rhizobiales (OCS116 clade, up to 9.3%) and Hyphomonadaceae (mainly *Hirshia* sp., up to 36%). PS diversity profiles displayed higher proportions of Saprospiraceae (up to 19%, mainly *Lewinella* sp.) and Planctomycetaceae (up to 6.3%) at the end of the experiment. Likewise, PLA mature biofilm was composed of Planctomycetaceae (up to 12%, mainly *Planctomyces* sp. and *Rhodopirellula* sp.) and Flavobacteriales (up to 7%, mainly *Luteibaculum* sp.) with increasing proportions over the course of the experiment.

The diversity of mature biofilms was clearly distinct among PHBV, PCL, and AA_144_OXO, with higher proportions of Alphaproteobacteria (mean = 36 ± 24%) compared to the non-biodegradable ones, followed by Gammaproteobacteria (mean = 29 ± 18%) and Flavobacteriia (mean = 11 ± 8.1%). PHBV is composed of Flavobacteriaceae (up to 26%, mainly *Maritimonas* sp.), Cellvibrionaceae (up to 15%, mainly *Marinicella* sp.) and Rhizobiales (up to 12%, OCS116 clade), especially at the end of the incubation. PCL and AA_144_OXO shared high proportions of Cellvibrionaceae (up to 23%) and Oceanospirillaceae (up to 52%, mainly *Pseudohongiella* sp.), but also specificities among Flavobacteriaceae, mainly *Aquibacter* sp. for PCL and *Gilvibacter* sp. for AA_144_OXO. PCL also comprised Hyphomonadaceae (up to 40%, mainly *Hirshia* sp. and *Hyphomonas* sp.), whereas AA_144_OXO showed noticeable proportions of Myxococcales (up to 23%, mainly Sandaracinacea family).

**FIGURE 5:**
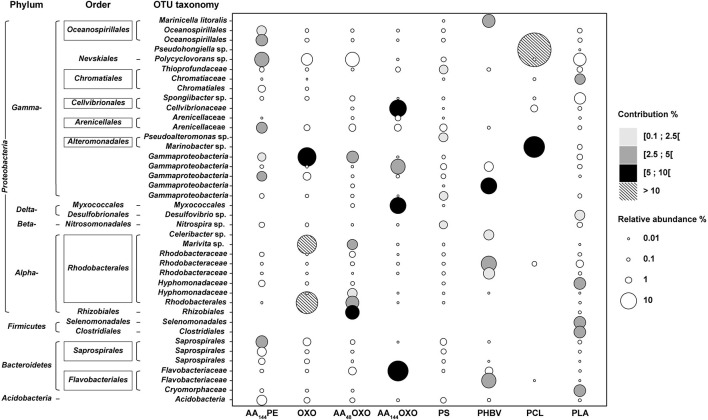
Bubble plot showing the relative abundance and the taxonomy of the OTUs contributing to a cumulated 30% of the dissimilarity between PE and the eight other plastic materials (AA_144_PE, OXO, AA_48_OXO, AA_144_OXO, PS, PHBV, PCL, and PLA) after 206 days of immersion in seawater (SIMPER analysis). Points are sized according to the relative abundance and colored by their contribution to dissimilarity.

SIMPER analysis highlighted 40 dominant OTUs contributing to a cumulative 30% of the dissimilarity between PE, used as control, and the other polymers after 206 days in seawater (Figure 5). Among non-biodegradable polymers, comparison between PE and AA_144_PE biofilms included six specific OTUs (Oceanospirillales, *Polycyclovorans* sp., Arenicallaceae, and Saprospirales). These OTUs shared low relative abundance and contribution, indicating a rather limited effect of artificial aging on PE. Limited specificities were also found on the other non-biodegradable materials when compared to PE control. AA_48_OXO biofilm exhibited five contributive OTUs, including two exclusive (affiliated to Rhizobiales and Hyphomonadaceae) and three shared with OXO (affiliated to Rhodobacterales, *Marivita* sp., and Gammaproteobacteria). PLA exhibited six specific OTUs contributing between 3.6 and 3.9% (Clostridiales, Selenomonadales, Cryomorphaceae, Hyphomodadaceae, Chromatiaceae, and *Desulfuvibrio* sp.). Note that the most abundant taxon found on PLA (*Polycyclovorans* sp., 5.7% in relative abundance) was not found as PLA-specific taxa by SIMPER analysis. PS hosted four specific OTUs (Pseudoalteromonas, Thioprofundaceae, Gammaproteobacteria, and *Nitrospira* sp.) but with low relative abundance (1.9 to 2.5%) and low contribution to dissimilarity with the PE control (1.4 to 2.2%).

Interestingly, the most distinct taxa were found among the biodegradable polymers (PCL, PHBV, and AA_144_OXO). PCL biofilm presented two highly abundant and contributive OTUs affiliated with *Pseudohongiella* sp. and *Marinobacter* sp. (52% and 19% in relative abundance, 26% and 9% contribution, respectively). A third PCL-specific OTU affiliated with Flavobacteriaceae was identified (8% contribution and 16% relative abundance). PHBV biofilm highlighted six specific OTUs with 1.8% to 5.2% of contribution, affiliated with Cellvibrionaceae, Bacteroidetes, Rhodobacteraceae, and Flavobacteraceae as well as *Marinicella litoralis* and *Celeribacter* sp. AA_144_OXO biofilm exhibited four very abundant (from 8 to 17%) and contributive (from 4.5 to 9.8%) OTUs, affiliated with Flavobacteriaceae (also present on PHBV and AA_48_OXO), Gammaproteobacteria, Cellvibrionaceae, and Myxococcales.

## Discussion

### Successive Phases of Biofilm Formation on Plastics

Both epifluorescence microscopy and flow cytometry confirmed the succession of primo-colonization, growth, and maturation phases of the plastics biofilm, as it has been described earlier (see review by Jacquin et al., 2019). Most of the previous studies were based on experiments conducted over a several-week period, mainly on conventional polymer types generally found in the ocean (mostly PE, PET, PVC, and PS) and our study brings new data on microbial colonization of biodegradable plastics in seawater.

Our results showed a rapid bacterial colonization of more than 5 × 10^3^ cells mm^–2^ on all material types, which is consistent with values found in studies focusing on the primo-colonization of plastic biofilms (Lee et al., 2008; Oberbeckmann et al., 2015). Interestingly, we found a significant difference in cell counts on PE or OXO after only 3 days of immersion in seawater as compared to same but artificially aged polymer(one order of magnitude higher for AA_144_PE, AA_48_OXO, and AA_144_OXO). Such differences may be explained by the change in surface properties accompanying the artificial aging by UV and temperature (Attwood et al., 2006). Material properties (crystallinity and crystal structure, hydrophobicity, roughness, glass transition temperature, melting temperature, and modulus of elasticity) indeed play a crucial role in the selection of bacterial community in the early stage of colonization (Pompilio et al., 2008). We also observed significantly higher cell counts on PS, PHBV, and PCL as compared to PLA or PE during the primo-colonization phase, but complete characterization of surface properties was not done in this study.

The growth phase of the biofilms was characterized by cell count increase (visible by flow cytometry) together with the production of extracellular matrix (visible by epifluorescence microscopy) after 10 days of immersion in seawater. The final expansion of the biofilm to maturity was visible from day 31 to day 206 on the non-biodegradable polymers (PE, AA_144_PE, OXO, AA_48_OXO, and PS), as well as for PLA. Interestingly, the biofilm maturity was not reached for the biodegradable polymers (PCL, PHBV) and for the AA_144_OXO that continue to increase even after 206 days of incubation in seawater. Few other long-term studies (> 6 months) have also shown limited differences in biofilm maturation on non-biodegradable polymers, i.e., PE, PP, PET, or polycarbonate (PC) (Webb et al., 2009; De Tender et al., 2017). Our results are consistent with another study underlining the continuous growth of PHBV biofilms in seawater (Dussud et al., 2018b), albeit limited to 45 days of immersion. Our results thus confirm this trend on PHBV but for much longer period (206 days) and extend this observation to PCL and AA_144_OXO. These results open new questions on the final expansion of the biofilms growing on biodegradable polymers in seawater and call for long-term incubation period in controlled conditions.

### Dynamics of Extra- and Intracellular Bacterial Activities on Plastics

This study presents pioneer results of intra- and extracellular activities of biofilms growing on various plastics. Extracellular enzymes are necessary to hydrolyze polymers and organic particles into small molecules (< 600 Da) that can cross the bacterial cell membranes *via* permeases for further intracellular degradation (Azam and Malfatti, 2007). Activities of hydrolytic enzymes such as protease, lipase, and glucosidase have already been examined for various marine particles including suspended particulate matter, marine snow, and sinking particles collected by sediment traps (Severin et al., 2016 and references cited there in), but not on plastic particles. So far, only one study evaluated the intracellular bacterial heterotrophic production on plastics (Dussud et al., 2018b). To the best of our knowledge, this is the first study to merge extra- and intracellular activities. Overall, we found that the measured extra- and intracellular activities gave the same trends, with higher activities found on biodegradable compared to non-biodegradable materials. This result underlines the complementary role of extra- and intracellular enzyme activities to decompose and transform polymers and organic molecules for further metabolization.

The primo-colonizers (day 3) were particularly active whatever the plastic types, as depicted by intracellular heterotrophic activity and also by extracellular aminopeptidase and lipase (not for b-glucosidase). Such activity peak may certainly be due to the utilization of organic matter present in the “conditioning film” that forms rapidly on hard surfaces placed in seawater (Loeb and Neihof, 1975; Dobretsov, 2010).

Thereafter, clear distinction could be made for non-biodegradable vs. biodegradable materials. Both inter- and extracellular activities decreased to remained low values from day 3 and until the end of the experiment on all the conventional materials (PE, AA_144_PE, OXO, and PS). Interestingly, a significant and consistent decrease was also observed on PLA, a polymer known as biodegradable under industrial composting conditions or degradable in human body (Pillai and Sharma, 2010), but not biodegradable under marine conditions (Karamanlioglu et al., 2017).

Intra- and extracellular activities (including b-glucosidase) remained stable or even increase until 206 days of immersion in seawater for the biodegradable polymers, with higher activities found for PCL and PHBV compared to AA_144_OXO. This appears consistent based on the intrinsic features of PCL and PHBV whose ester linkages will be degraded by hydrolytic enzymes such as lipases. The potential activity of plastic biodegradation in seawater is determined by several standard tests based on biomass and respirometric activity measurements (mainly CO_2_ production) under laboratory conditions during 3 months (ASTM D6691-09), 6 months (ASTM D7473-12), or 24 months (ISO 18830, ISO 19679, ASTM D7991-15). Other activity tests can be considered as alternative methods, such as tests based on ATP measurements (Fontanella et al., 2013) or bacterial heterotrophic production (Jacquin et al., 2021). We are aware that intra- and extracellular bacterial activities under natural conditions may be related to processes other than plastic biodegradation (biodegradation of organic matter and cross-feeding in the biofilm) (Pande et al., 2014), but this study provides a set of congruent results that, although not definitely compelling, gives concrete hints for effective biodegradability in seawater (Jacquin et al., 2021).

### Microbial Community Dynamics in Seawater and for Biodegradable and Non-biodegradable Plastics

Illumina MiSeq sequencing provided a set of complementary information to better understand the dynamics of bacterial abundance and activities on plastic described above, including (i) a clear distinction between communities living on plastics and the surrounding seawater, (ii) contrasted communities on biodegradable and non-biodegradable materials after 7 months of immersion in seawater, which allowed to (iii) describe new potential candidates for the biodegradation of PCL, PHBV, and artificially aged OXO in natural seawater.

First, cluster analysis showed significant distinction between bacterial communities living on plastics when compared to the free-living and organic particle-attached fractions. Such observation is now a common feature observed in the oceans worldwide (Wright et al., 2021) that reinforce the originality of the “plastisphere” compared to other microorganisms living in the surrounding seawater. In our study, we noticed that organic particle-attached communities formed a separate group that was more similar to the plastisphere compared to the free-living fraction, as already mentioned on PET drinking bottles and glass substrates in seawaters (Oberbeckmann et al., 2016). These authors suggested that the similarity between organic particle-associated and plastic biofilms may be explained by the presence of non-specific surface-colonizing microbes that represent another part of the diversity around the core plastisphere microbiome. Indeed, surface attachment capabilities of microorganisms can be triggered by organelles (e.g., flagella, pili) (Rogers et al., 2020), hydrophobicity of the outer membrane (e.g., modifications of the lipopolysaccharidic composition of some bacteria allowing better interactions with hydrophobic substances) (Krasowska and Sigler, 2014), or synthesis of surfactants (e.g., higher ability of *Lysinibacillus fusiformis* to degrade polyethylene in the presence of biosurfactants) (Mukherjee et al., 2016). We also found clear dissimilarities between the bacterial communities in the free-living fraction (dominated by Alphaproteobacteria, mainly SAR11) and the organic particle-attached fraction (dominated by Gammaproteobacteria), which is also a common pattern in seawaters (Li et al., 2021). Both free-living and organic particle-attached communities remained relatively stable during the entire course of the experiment, with classical values found in the Mediterranean seawaters in terms of abundance, heterotrophic bacterial production, and extracellular activities (Severin et al., 2016). These results validated our capability to maintain realistic conditions for up to 206 days in an experimental setup renewed with natural seawater every 30 min.

Second, we observed a succession of communities with clear dissimilarities between biodegradable and non-biodegradable materials after 7 months of immersion in seawater. Such dissimilarities were not found during the primo-colonization (day 3) and the growth of the biofilm (day 10) that showed distinct but close communities whatever the plastic type. Primo-colonizers (day 3) together with bacteria on the growth phase of the biofilm (day 10) belonged to Gammaproteobacteria (mainly *Alteromonadaceae*, *Oceanospirillaceae*, and *Alcanivoracaceae*), which is in accordance with previous studies (Dussud et al., 2018a; Cheng et al., 2021). Under our conditions, significant dissimilarities were observed after 31 days, which corresponds to the maturity of biofilms for non-biodegradable materials on the one hand, and to the continuous growth with high extra- and intracellular activities for biodegradable materials on the other hand. After 31 to 74 days of immersion in seawater, biofilms on PCL and PHBV strongly differed from the mature biofilms of other plastics. AA_144_OXO also formed a distinct cluster after 116 days. This is the first time such comparison has been reported for long-term studies, as the only one study comparing bacterial abundance, activity, and diversity on biodegradable materials ended their experiment at the beginning of the biofilm maturity, i.e., after 45 days of immersion in seawater (Dussud et al., 2018a). Most of the other long-term studies over a period of more than 6 months restricted their analysis on bacterial diversity colonizing non-biodegradable polymers, such as PE, PP, PET, and PC (Webb et al., 2009; De Tender et al., 2017). We emphasized here that further studies may take into account not only the bacterial diversity living on plastics, but also the extra- and intracellular activities of the biofilm that will provide new insights into the functional role played by the plastisphere in seawaters.

Third, we describe new potential candidates for the biodegradation of PCL, PHBV, and artificially aged OXO in natural seawater, based on the congruent results of biomass increase together with high extra- and intracellular activities. We used similarity percentage analysis (SIMPER, Ghiglione et al., 2012) to identify OTUs primarily responsible for dissimilarities between pairs of PE versus PCL, PHBV, and AA-OXO after 206 days of incubation in seawater. A clear trend was observed with biodegradable polymers being dominated by a low number of highly abundant contributive OTUs when compared to non-biodegradable polymers, suggesting a specialization of the bacterial communities, providing more information to the contrasted results obtained so far, not allowing to answer whether plastics really select for specific microbial communities (Wright et al., 2020). The most important OTUs that influenced the dissimilarity between PCL and PE were *Pseudohongiella* sp. and *Marinobacter* sp. These two genera have never been listed as PCL degraders, but the first was found highly abundant after crude oil pollution (Valencia-Agami et al., 2019; Peng et al., 2020) and the second contains well-known hydrocarbonoclastic species (Grimaud et al., 2012; Cui et al., 2016), which is consistent with the petroleum nature of the polymer. Another contributing OTU was affiliated with Flavobacteriaceae that were already shown as potential PCL degraders (Lopardo and Urakawa, 2019) or capable of using complex carbon sources (Rodriguez-Sanchez et al., 2016). We also identified putative PHBV degraders, some of them being already highlighted in similar studies, such as Bacteroidetes and others presenting the enzymatic machinery for PHA degradation, such as Celeribacters (Volova et al., 2016). We also found *Marinicella litoralis* that has never been described as a potential PHBV degrader to date (Romanenko et al., 2010; Shoji et al., 2014), as well as Rhodobacteraceae that was abundant in all material types with known ability to form biofilm on plastics and to degrade complex organic matter (Kessler et al., 2011; Flood et al., 2018). Finally, putative degraders of artificially aged OXO (AA_144_OXO) belonged to Gammaproteobacteria, Cellvibrionaceae, Myxococcales, and Flavobacteriaceae, which are classically observed on the plastisphere of various material types found in seawaters including PE (Zettler et al., 2013), PET (Oberbeckmann et al., 2016), and PVC (Pinto et al., 2019). Members of Myxococcales and Flavobacteriaceae presented high hydrolytic enzymes excretion and complex biopolymers degradation capacities (Shimkets et al., 2006; Amaral-Zettler et al., 2020) together with hydrocarbon degradation capabilities (Pollet et al., 2018; Gutierrez, 2019). We are aware that the description of putative OTUs involved in the biodegradation of PCL, PHBV, and AA-OXO are sometimes limited to the poor taxonomic identification based on 16S rRNA metabarcoding; however, it opens new routes for further studies to delve deeper into the understanding of the biodegradability of these materials in seawaters.

## Concluding Remarks

Through the analysis of a broad set of microbial parameters (abundance, diversity, and activities) to describe the evolution of the marine biofilms growing on six different plastic types during a long-term experiment, we demonstrated clear evidence of biodegradation on PCL, PHBV, and artificially aged OXO in seawater. Signs of biodegradation were visible after 1 month and until the end of the experiment, with significant increases of the biofilm biomass, of the intra- and extracellular activities, together with drastic changes in bacterial diversity. In contrast, we found no sign of biodegradation for PE, PS, and PLA under our experimental conditions, where the biofilms became mature after 1 month and remained relatively stable until the end of the experiment. We encourage the recording of these microbial parameters for a comprehensive connection between biofilm formation and polymer degradation when evaluating the plastic biodegradation in seawater, which is currently not taken into account in the current standards (mainly based on respirometry activities and generally performed under laboratory conditions). Our study opens also new routes for further studies on biodegradation mechanisms, by providing a list of putative OTUs that may serve as model organisms potentially involved in the biodegradation of PCL, PHBV, and artificially aged OXO under natural seawater conditions.

Finally, it is noteworthy that the bio-based polymer PLA showed no signs of biodegradation under our experimental conditions, whereas the fossil-based polymer PCL showed significant evidence of biodegradation. This reinforced the distinction that has to be made between biobased and biodegradable polymers but also the different certification schemes for biodegradation in contrasted environments (compost, freshwater, and seawater) when considering an alternative to conventional fossil-based plastics.

## Data Availability Statement

The datasets presented in this study can be found in online repositories. The names of the repository/repositories and accession number(s) can be found below: https://www.ebi.ac.uk/metagenomics/, PRJEB37662.

## Author Contributions

CD, A-LM, SB, and J-FG have conceived and designed the study. CD, ML, and CO carried out all the experiments and acquired the data. SB and BE provided the equipment. CD, CO, LP, GD, A-LM, VB, and J-FG analyzed and interpreted the data. CO, CD, VB, and J-FG drafted the manuscript. LP, GD, ML, BE, GB, AT, A-LM, and SB revised the manuscript and approved the version of the manuscript to be published. All authors contributed to the article and approved the submitted version.

## Conflict of Interest

The authors declare that the research was conducted in the absence of any commercial or financial relationships that could be construed as a potential conflict of interest.

## Publisher’s Note

All claims expressed in this article are solely those of the authors and do not necessarily represent those of their affiliated organizations, or those of the publisher, the editors and the reviewers. Any product that may be evaluated in this article, or claim that may be made by its manufacturer, is not guaranteed or endorsed by the publisher.
